# Clinical and genetic features of luscan-lumish syndrome associated with a novel de novo variant of *SETD2* gene: Case report and literature review

**DOI:** 10.3389/fgene.2023.1081391

**Published:** 2023-01-27

**Authors:** Yanqing Zhang, Haozheng Zhang, Wei Wu, Dong Wang, Yuqiang Lv, Dongmei Zhao, Lingxiao Wang, Yi Liu, Kaihui Zhang

**Affiliations:** ^1^ Pediatric Healthcare Institute, Children’s Hospital affiliated to Shandong University (Jinan Children’s Hospital), Jinan, Shandong, China; ^2^ Pediatric Research Institute, Children’s Hospital affiliated to Shandong University (Jinan Children’s Hospita), Jinan, Shandong, China; ^3^ Department of General Pediatric, Children’s Hospital of Fudan University, National Children’s Medical Center, Shanghai, China

**Keywords:** developmental delay, luscan-lumish syndrome, SETD2, gene mutation, tall stature, macrocephaly

## Abstract

**Introduction:** Luscan-Lumish syndrome (LLS) is currently recognized as a rarely-observed condition featured with overgrowth, macrocephaly, obesity, type I Chiari malformation, and linguistic retardation. So far, there have been only a few LLS cases registered worldwide, but with none of them reported from China. To acquire a deeper understanding on the clinical and genetic features of this disease, a Chinese boy with LLS caused by a heterozygous variant in SETD2 gene was investigated in the present study.

**Methods:** The patient was clinically examined and the medical history of his family was collected. Genetic testing was performed to determine the genetic etiology.

**Results:** The proband was a boy aged 5-year-7-month-old, who was referred to our hospital due to “being a slow learner in kindergarten”. The child had a history of delayed motor and language development in comparison to his peers. After admission, physical examination revealed tall stature and macrocephaly as the major manifestation, in addition to a relatively lower rating in intelligence assessment as well as abnormal MRI images showing a slightly shorter corpus callosum accompanied by a mildly thinner corpus callosum body. Whole exome sequencing (WES) revealed a heterozygous c.2514_2516delTAG (p.Ser838del) variant in SETD2 gene, which was subsequently identified as a novel *de novo* variant. According to the standardized genetic variant classification published by the American College of Medical Genetics and Genomics (ACMG), the variant, with a pathogenicity analysis result indicating PS2 + PM2_Supporting + PM4, was determined to be likely pathogenic. Through literature review, the clinical phenotypes of the 15 LLS cases were summarized, including 8 cases of overgrowth (53%), 13 cases of macrocephaly (87%), 11 cases of developmental delay (73%), 8 cases of autism (53%), and 7 cases of special facial features (47%). Besides, abnormal craniocerebral MRI findings were noticed in 7 cases. Despite that the mutation sites of the 15 patients varied from case to case, they showed a uniformly distributed pattern throughout the whole SETD2 gene, including 5 missense mutations, 5 frameshift mutations and 5 non-sense mutations.

**Conclusion:** LLS, not having been recognized till recent years, is identified as an autosomal dominant syndrome triggered by SETD2 gene mutation. As the first report of LLS in China, the case in our study was proved to be associated with a unique type of SETD2 gene mutation that has never been reported previously, which is believed to enrich the mutation spectrum of SETD2 gene and also, deepening the clinicians’ understanding on the disease.

## Introduction

Luscan-Lumish syndrome (LLS, OMIM: 616831), as a rare condition characterized by overgrowth, macrocephaly, obesity, type Ⅰ Chiari malformation, and linguistic retardation, has been recognized over the recent years as an autosomal dominant genetic condition caused by SETD2 gene variant (van Rij et al., 2018; Suda et al., 2021). Locating in 3p21.31, the SETD2 gene consists of 23 exons and 22 introns, with a length of about 147 KB. The histone methyltransferases (HMTs) encoded by SETD2 gene, as a critical epigenetic regulator, plays a key role in multiple biological processes such as regulating histone methylation, gene transcription, maintaining gene stability and remodeling the epigenome and (epi) cytoskeleton (Park et al., 2016; Walker and Burggren, 2020; Koenning et al., 2021; Liu et al., 2021; Seervai et al., 2021). To date, few cases about this disease have been reported worldwide, and none has ever been recorded in China. In the present study, by next-generation sequencing on a proband with a major manifestation of delayed language development, recognition lag, hypomegasoma, and macrocephaly, we successfully identified the genetic etiology of this disease.

## Case description

The male proband aged 5 years and 7 months old was admitted to our hospital in November 2020 due to “being a slow learner in kindergarten”. The child, once hospitalized for premature birth for half a month, was the third fetus and the third birth of the mother who showed no abnormalities during pregnancy but was admitted for cesarean section at 36 weeks owing to intrauterine distress. His motor and language development were delayed compared with children of the same ages, as demonstrated by not being able to walk and say some simple words till about 1.5 years of age. After a long period of stagnation in language progress, his vocabulary gradually expanded at about 5 years of age. When the boy was admitted, he could only speak four to five words in short sentences. In the middle class of his kindergarten, he was noticed to be a slow learner with a poor ability of language expression and have little communication with his classmates. At home, besides being capable of responding to his family and expressing his needs with simple words, the boy could also share attention with them. No obvious stereotyped behavior was observed except for his particular obsession with Altman, as well as the likings of shaping plasticine and painting. On examination, the child was measured as 124.5 cm (>+2SD) in height and 54 cm (>+2SD) in head circumference, without unusual facial features or any abnormalities observed in cardiopulmonary auscultation and other physical examinations. Behavior evaluation in the clinic revealed normal eyes contact, as well as the ability to call his mother and to ask and answer simple questions.

Neither history of major physical diseases nor family genetic history was reported in the family except the proband. The patient had two elder sisters, the normally-developed 16-year-old sister being a freshman in senior high school with a good academic performance, and the 7-year-old sister in grade one with an average school record.

Gesell development schedules (GDS) indicated a score of 47 in adaptability, 45 in large motor movement, 46 in fine motor movement, 33 in language, and 39 in personal-social development. No abnormalities were found in blood routine, thyroid function or biochemical tests. Hearing test also showed no anomalies. Craniocerebral magnetic resonance imaging (MRI) revealed a slightly shorter corpus callosum with a mildly thinner corpus callosum body (Supplementary Figure S1).

## Methods

### Sample collection

2 ml of peripheral blood was collected respectively from the child and his parents for chromosome karyotype analysis and next-generation sequencing for whole exome.

### Chromosome karyotyping

2 mL of peripheral blood was collected under sterile conditions, which, after being anticoagulated with heparin, was inoculated into lymphocyte medium and then cultured at 37°C for 72 h to collect cells, followed by a G-banding process for analysis using the image analysis system (Leica, United States).

### Targeted next-generation sequencing and sanger sequencing

2 ml of peripheral blood samples, after collected respectively from the child and his parents, was anticoagulated with EDTA. DNA was extracted from the samples using TGuide Blood genomic DNA extraction kit. Following the construction of the genomic library, the whole exome sequencing (WES) kit (MyGenostics Inc, Beijing, China) was employed to capture the exon region as well as 50 bp of its upstream and downstream region. Thereafter, paired-end sequencing was performed in the captured region by Illumina HiSeq X ten, a high-throughput sequencing platform with a read length of 150 bp.

Sanger sequencing was applied to verify the mutations found by WES. The primer sequences used in this test for the mutation, c.2514_2516delTAG, were 5′-TGA​ACT​AGT​GCT​ACC​GAT​GCT-3′ in the forward primer and 5′-TGA​TAG​TGT​GAC​TGG​ATC​GGA-3′ in the reverse primer.

### Data screening and bioinformatics analysis

After sequencing of the target region, the adapters and low-quality data were removed from the data. BWA (http://bio-bwa.sourceforge.net/) was applied to align the collected data with the reference genome (hg19) for statistical analysis of the parameters, including the sequencing depth, homogeneity, and probe specificity. Based on the obtained data, GATK (https://software.broadinstitute.org/gatk/) was then used to detect the DNA polymorphism, and to statistically analyze the single nucleotide polymorphisms (SNPs) as well as the insertions and deletions (InDels). Moreover, all the SNPs and InDels were annotated by ANNOVAR (http://annovar.openbioinformatics.org/en/latest/) with access to multiple databases such as 1000 genome, ESP6500, dbSNP, EXAC, Inhouse (MyGenostics), HGMD, SIFT, PolyPhen-2, MutationTaster, and GERP++. Pathogenicity was graded according to American College of Medical Genetics and Genomics (ACMG) guidelines, followed by the conservative analysis using Unipro UGENE. Conservation analyses among diverse species were performed using ClustalX software.

## Results

### Chromosome karyotyping

Karyotype analysis of the child indicated 46, XY without any abnormalities detected. There was no abnormality, so we did not show the karyotype map.

### Targeted next-generation sequencing and sanger sequencing

**FIGURE 1:**
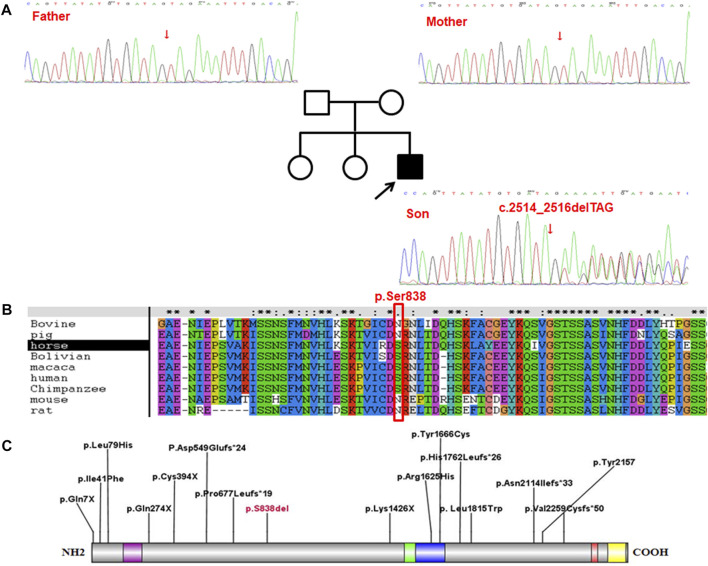
The proband carried the c.2514_2516delTAG (p.Ser838del) mutation in SETD2 gene. **(A)**, The results of Sanger sequencing on **(C)**2514_2516delTAG (p.Ser838del) mutation in SETD2 gene in the proband and his family. **(B)**, Conservative analysis at site p. Ser838 in multiple species. **(C)**, Distribution of the variant sites in 16 LLS patients.

The results demonstrated a higher than 98.47% coverage of the target genes which had a sequencing depth of more than 20×, with an average sequencing depth of 360.5×, indicating that the quality of the data was in accordance with the standard as required. After sequencing and screening, a heterozygous variant, c.2514_2516delTAG (p.Ser838del), was spotted in the SETD2 gene of proband, which, identified as a codon mutation, subsequently resulted in an alteration in the composition of the amino acids (p.Ser838del). In contrast, Sanger sequencing revealed no mutation at this site of both his parents (Figure 1).

### Pathogenicity analysis of the variant sites

Pathogenic analysis of the detected variants was carried out according to the ACMG guidelines (2015). The variant c.2514_2516delTAG (p.Ser838del) in SETD2 gene was identified as a *de novo* variant with a classification of strong pathogenicity (PS2). This mutation not having been detected in the databases of both normal population and the local normal population, the evidence was thus classified as PM2_Supporting. Furthermore, the loss of the termination codon for the protein encoded by the gene, which was caused by this variant, consequently led to the alteration in the protein length. Therefore, the pathogenic evidence was categorized as PM4 level. Considering all the factors as described above (PS2 + PM2_Supporting + PM4), this variant was eventually identified as being likely pathogenic. Combining clinical features and genetic testing, We finally diagnosed the patient as Luscan-Lumish syndrome.

### Bioinformatics analysis

Conservative analysis revealed that Serine at site 838 (p.Ser838) was partly conserved in multiple species (Figure 1). However, either serine or asparagine at the site 838 is neutral amino acid, and no loss is found at the site in multiple species. Therefore, it is speculated that the loss of amino acids at site 838 (p.Ser838) affects the protein function, which needs further experimental verification.

## Discussion

LLS, being an autosomal dominant syndrome as a result of SETD2 gene mutation, has only been recognized till 2012 (8-11). Since then, the syndrome has been officially named Luscan-Lumish syndrome (LLS), also known as “SETD2-related overgrowth syndrome” (van Rij et al., 2018). To date, LLS has manifested a diversity of clinical phenotypes, among which the commonly-observed phenotypes included postnatal overgrowth, macrocephaly, developmental delay and autism spectrum disorder (ASD) (Table1). Including our case, 56% of the cases were taller than children of the same age and sex in childhood, which has been speculated involving a mechanism relevant to GH-dependent enhancement of STAT5b signaling (Suda et al., 2021). However, according to the data reported in the literature, the patients who were followed up to adulthood did not finally have a high stature compared with their peers, which led to another speculation that the high stature in childhood might be related to the advanced bone age of these children (Luscan et al., 2014). As for our case, he was measured a height of 124.5 cm (>+2SD) at the age of 5 years and 7 months, which was higher than children of the same age, and the final height was also needed to monitored.

**TABLE 1:** Summarization for genetic variants and clinical phenotypes of Luscan-Lumish syndrome.

Number of the cases	Gender	Age (y)	Alteration in amino acids	Variants	Chromosomal location	Genetic origin	Overgrowth	Macrocephaly	Unusual facial features	Developmental delay
1	M	4	p.Cys394X	c.1182T>A	47164944	P	+	+	Na	+
2	M	4	p.Gln7X	c.19C>T	4720539	M	-	-	Na	-
3	M	13	p.Ile41Phe	c.121A>T	47166005	*de novo*	-	+	Na	na
4	F	10	p.Asn2114Ilefs*33	c.6341delA	47098932	*de novo*	na	+	Na	+
5	M	26	p.Leu1815Trp	c.5444T>G	47125826	*de novo*	+	+	+	+
6	F	23	p.Gln274X	c.820C>T	47165306	*de novo*	+	+	+	+
7	F	15	p.Pro677Leufs*19	c.2028delT	47164098	*de novo*	-	+	-	+
8	M	12	p.His1762Leufs*26	c.5285_5286delAC	47127795-47127797	*de novo*	+	+	+	+
9	M	4.5	P.Asp549Glufs*24	c.1647_1667delinsAC	47164458-47164479	*de novo*	-	+	+	+
10	F	23	p.Val2259Cysfs*50	c.6775delG	47098498	*de novo*	+	+	+	+
11	F	3	p.Lys1426X	c.4276A>T	47161850	*de novo*	+	+	+	-
12	F	5	p.Arg1625His	c.4874G>A	47144879	*de novo*	-	+	-	+
13	F	8	p.Tyr2157X	c.6471 T>A	47098803	*de novo*	+	+	+	+
14	M	10	p.Tyr1666Cys	c.4997 A>G	47142966	*de novo*	-	-	+	+
15	M	20	p.Leu79His	c.236T>A	47165890	*de novo*	+	na	+	-
The proband in the study	M	5	p.Ser838del	c.2514_2516delTAG	47163609-47163612	*de novo*	+	+	-	+

Number of the Cases	ASD	Behavioral disorders	Seizures	Otitis media	Gastroesophageal reflux	Hypomyotonia	Changes in craniocerebral images	Advanced bone age	References
1	+	na	na	+	+	Na	na	na	O'Roak et al. (2012)
2	+	-	na	+	na	na	na	na	O'Roak et al. (2012)
3	+	na	na	+	+	na	na	na	O'Roak et al. (2012)
4	+	na	+	+	+	+	na	na	O'Roak et al. (2012)
5	na	-	na	na	na	na	na	+	Luscan et al. (2014)
6	na	+	na	na	na	+	+	na	Luscan et al. (2014)
7	+	+	+	+	na	+	+	na	Luscan et al. (2014)
8	na	na	na	+	na	na	na	+	Tlemsani et al. (2016)
9	+	na	na	na	na	na	-	-	van Rij et al. (2018)
10	+	+	na	+	na	-	+	+	van Rij et al. (2018)
11	na	+	-	+	na	na	+	na	Marzin et al. (2019)
12	-	+	na	na	na	na	+	na	Marzin et al. (2019)
13	+	+	na	na	na	na	-	na	Marzin et al. (2019)
14	na	na	+	na	na	na	+	na	Marzin et al. (2019)
15	-	-	-	na	na	-	+	na	Suda et al. (2021)
The proband in the study	-	-	-	-	-	-	+	na	the reported cased

Note: na, not available.

Macrocephaly has been recognized as another major phenotype of LLS, as demonstrated by the available data from literature review showing that 13 out of the 15 cases (87%) had a head circumference greater than 97% of children of the same age and sex, which was also a feature consistent with our case (Table1). Among these patients, abnormal craniocerebral MRI findings, though with a great heterogeneity between individuals, were spotted in 7 cases (Table1). In our case, the craniocerebral MRI image displayed a slightly shorter corpus callosum accompanied by a mildly thinner corpus callosum body, which was a finding not reported in any of the previous cases of LLS. Otherwise, about 14%–18% of individuals with autism have head circumferences above the 97th percentile (Stevenson et al., 1997). Thus, it is necessary to distinguish between Luscan-Lumish syndrome and autism, and the next-generation sequencing technology is a good way to detect these diseases, especially for LLS. ASD and ID are also considered as major phenotypes of LLS syndrome. Among all the cases that we reviewed, eight children were diagnosed with ASD and 11 children with ID. It has been speculated that SETD2 gene variant is likely to be a candidate gene causing ASD or ID (O'Roak et al., 2012; Tlemsani et al., 2016; Lumish et al., 2015). Also, our case was presented to the hospital because of learning difficulties, and he did not correspond to the diagnostic criteria for ASD. However, the excessive height and head circumference of the child failed to draw much attention from the parents, which suggested the necessity and significance for clinical practitioners to carry out systematic physical examinations for children with developmental delay, especially genetics testing aiming at etiological analysis, for those with abnormal symptoms and signs.

Among the 15 reported LLS, the mutations showed a uniform distribution throughout the whole SETD2 gene, including 5 missense mutations, 5 frameshift mutations and 5 non-sense mutations (Figure 1). As a result, these variants led to malfunction of the proteins encoded by the SETD2 gene, the p. Arg1625Cys and p. Tyr1666Cys were located in the catalytic domain, the other three missense variants were distributed between the functional domains; The p. Leu1815Trp variant was spotted in a patient similar to Sotos syndrome (Luscan et al., 2014); The p. Ile41Phe variant was identified to be associated with isolated ASD (O'Roak et al., 2012); the p. Leu79His variant was detected in a 20-year-old male patient with LLS (Suda et al., 2021); Furthermore, SETD2 and NSD1 proteins, as two methyltransferases of histone H3 lysine 36 (H3K36) in nucleosomes, are involved in some specific biological processes such as transcription elongation, RNA processing and DNA repair. Functional studies on p. Arg1625Cys revealed that complete loss of H3K36me3 was associated with the loss of protein function (Hacker et al., 2016; Marzin et al., 2019). However, due to the limited number of patients having been investigated, definitive conclusions on the genotype-phenotype correlation cannot be drawn at this time.

A number of studies have reported that variants in somatic SETD2 gene are related to the occurrence of a variety of tumors, especially renal cell carcinoma, pulmonary adenocarcinoma, enteropathy-associated T cell lymphoma, chronic lymphocytic leukemia, and gastrointestinal stromal tumors (D'Avella et al., 2020; Tessema et al., 2018; Moffitt et al., 2017; Parker et al., 2016; Huang et al., 2016). Compared with the general population, patients with overgrowth syndrome tend to register a slightly increased prevalence of tumors, with studies reporting a 7% risk of developing tumors in Beckwith-Wiedemann syndrome and a 3%-5% elevation in the risk of a wide range of tumors in Sotos and Weaver syndromes (Tatton-Brown and Rahman, 2013; Villani et al., 2017; Brioude et al., 2019). So far, only a solitary case with meningioma was detected by craniocerebral MRI among all the reported patients with LLS syndrome. Given that most of the patients were children or young adults, long-term follow-ups should be performed to assess the potential risk of neoplasia.

The proband in our study presented with developmental delay, tall stature and macrocephaly, in combination with an abnormal craniocerebral MRI imaging, which was considered to be consistent with the main phenotypes of LLS. Subsequently, a heterozygous c.2514_2516delTAG (p.Ser838del) mutation in the SETD2 gene was identified, which, as a codon mutation, consequently resulting in the malfunction of the amino acid encoded by the gene. The present study, as the first report of LLS syndrome in China where the mutation of SETD2 gene has never been previously delineated, is believed to be a contribution to the enrichment of the mutation spectrum of SETD2 gene as well as the improvement of clinicians’ further understanding on this disease.

## Data Availability

The original contributions presented in the study are publicly available. This data can be found here: https://www.biosino.org/node/project/detail/OEP003733.

## Supplementary material

SUPPLEMENTARY FIGURE S1 Brain MRI. The left cerebral hemisphere is slightly larger, the corpus callosum is slightly shorter, the shape of the lateral ventricle is not good, frontal sinusitis. Arrows show corpus callosum.
