# Supplementary figures and images for: Clinical and genetic features of luscan-lumish syndrome associated with a novel de novo variant of SETD2 gene: Case report and literature review
Source: Front Genet. 2023 Jan 27;14:1081391. doi: 10.3389/fgene.2023.1081391 (PMC9911649; doi:10.3389/fgene.2023.1081391)

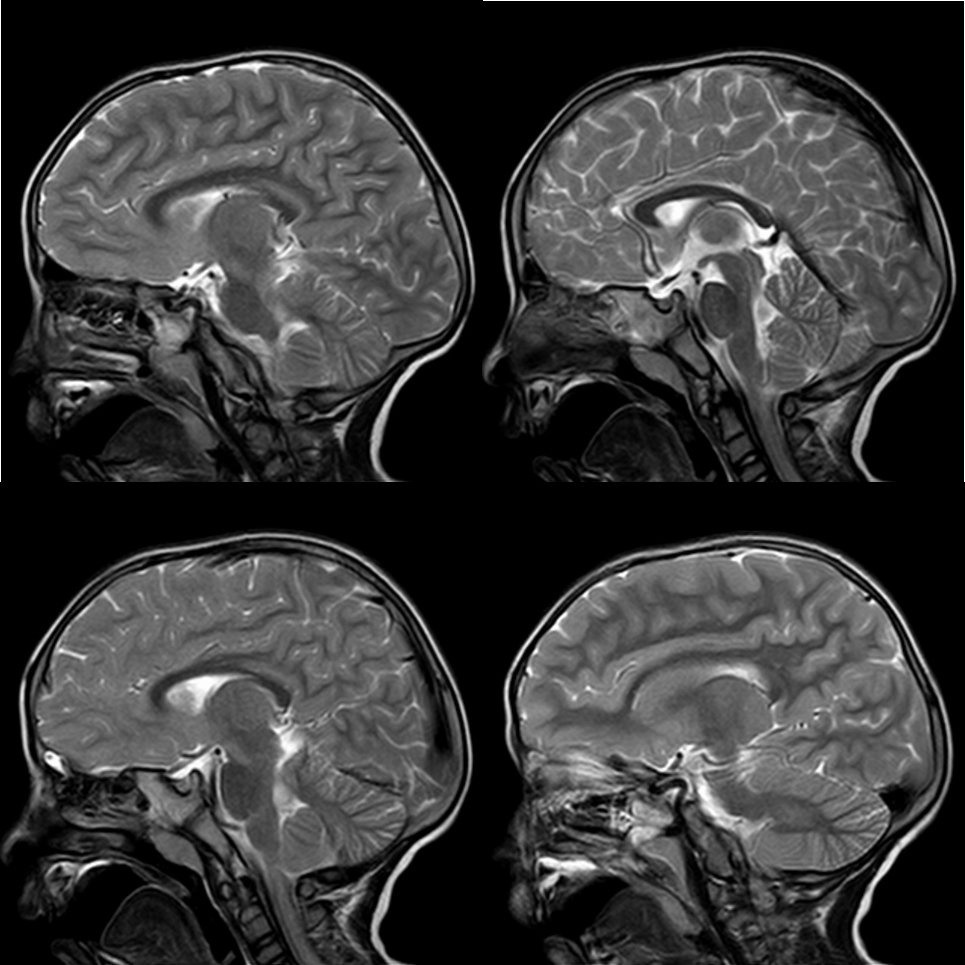

Supplement: Supplementary file 1 [file Image1.TIF]
